# Host Immune Selection of Rumen Bacteria through Salivary Secretory IgA

**DOI:** 10.3389/fmicb.2017.00848

**Published:** 2017-05-12

**Authors:** Janelle M. Fouhse, Luke Smiegielski, Melanie Tuplin, Le Luo Guan, Benjamin P. Willing

**Affiliations:** Department of Agricultural, Food and Nutritional Science, University of Alberta, EdmontonAB, Canada

**Keywords:** rumen, saliva, secretory-IgA, microbiota, cattle

## Abstract

The rumen microbiome is integral to efficient production in cattle and shows strong host specificity, yet little is known about what host factors shape rumen microbial composition. Secretory immunoglobulin A (SIgA) is produced in large amounts in the saliva, can coat both commensal and pathogenic microbes within the gut, and presents a plausible mechanism of host specificity. However, the role salivary SIgA plays in commensal bacteria selection in ruminants remains elusive. The main objectives of this study were to develop an immuno-affinity benchtop method to isolate SIgA-tagged microbiota and to determine if salivary SIgA preferentially binds selected bacteria. We hypothesized that SIgA-tagged bacteria would differ from total bacteria, thus supporting a potential host-derived mechanism in commensal bacterial selection. Whole rumen (*n* = 9) and oral secretion samples (*n* = 10) were incubated with magnetic beads conjugated with anti-secretory IgA antibodies to enrich SIgA-tagged microbiota. Microbial DNA from the oral secretion, whole rumen, SIgA-tagged oral secretion, and SIgA-tagged rumen was isolated for amplicon sequencing of V1–V3 region of 16S rDNA genes. Whole rumen and oral secretion had distinctive (*P* < 0.05) bacterial compositions indicated by the non-parametric multidimensional scaling plot using Euclidean distance metrics. The SIgA-tagged microbiota from rumen and oral secretion had similar abundance of Bacteroidetes, Actinobacteria, Fibrobacter, candidate phyla TM7, and Tenericutes and are clustered tightly. Composition of SIgA-tagged oral secretion microbiota was more similar to whole rumen microbiota than whole oral secretion due to enrichment of rumen bacteria (Lachnospiraceae) and depletion of oral taxa (*Streptococcus, Rothia*, Neisseriaceae, and Lactobacillales). In conclusion, SIgA-tagged oral secretion microbiota had an increased resemblance to whole rumen microbiota, suggesting salivary SIgA-coating may be one host-derived mechanism impacting commensal colonization. Further studies, to explore the variations in antibody affinity between different animals as a driver of microbial composition are warranted.

## Introduction

The rumen of cattle hosts a dynamic and complex microbiome that is essential for the conversion of indigestible plant biomass into useable energy substrates. The composition of rumen microbiota can be affected by many exogenous factors, especially diet, and changes in rumen microbiome have been reported to be associated with host production and metabolic related phenotypes such as feed efficiency (Myer et al., 2015), methane emission (Ross et al., 2013), and ruminal acidosis (Khafipour et al., 2009). Rumen transplant has demonstrated that the mature rumen microbiome is resilient to exogenous bacteria (Weimer et al., 2010), indicating strong host mechanisms work to retain specific bacteria while eliminating others. However, the intrinsic host-derived mechanisms working to retain or eliminate rumen commensal microbiota remains somewhat elusive.

The mucus barrier, antimicrobial peptides, and secretory immunoglobulin A (IgA) production are all known host-derived mechanisms that exist to help maintain the symbiotic relationship between the microbiome and host (Bunker et al., 2015). Secretory-IgA (SIgA) is the most abundant immunoglobulin produced in all animals, secreted at the epithelial membranes of all mucosal surfaces including the mouth (Macpherson et al., 2012). SIgA has been detected in bovine saliva and is found to be the most prevalent immunoglobulin (Mach and Pahud, 1971). SIgA plays an interesting role in microbial symbiosis by stimulating both immune protection and preservation of immune homeostasis (Cerutti et al., 2011). SIgA can inhibit pathogenic bacteria colonization by interfering with epithelial binding, targeting them for removal through T-dependent mechanisms (Brandtzaeg, 2010; Cerutti et al., 2011).

Secretory-IgA has been shown to coat 40–80% of small intestinal commensal bacteria in mice (Bunker et al., 2015), 24–74% of anaerobic bacteria in human feces, and 20% of commensal bacteria within the rumen of calves (Tsuruta et al., 2012), potentially indicating SIgA-coating of bacteria may also be fundamental to governing commensal microbiota composition (Van Der Waaij et al., 1996; Cebra et al., 1998). However, to date, no one has characterized the composition of salivary SIgA-tagged bacteria in comparison to total rumen microbiota in adult cattle. Based on previously established relationships between SIgA and commensal microbiota within the gut, we hypothesized that salivary SIgA is a potential host-derived mechanism selecting commensal rumen bacteria. We expected that SIgA-tagged bacteria would differ from total bacteria. Current methodology to detect SIgA-tagged microbiota is limited to the use of fluorescence-activated cell sorting flow cytometry (Palm et al., 2014; Bunker et al., 2015; Kau et al., 2015). Therefore development of an effective immune-affinity benchtop method to separate SIgA-tagged bacteria from whole rumen or oral secretion samples for rapid characterization of SIgA-tagged microbiota was deemed essential. The objectives of this study were to develop a novel methodology to separate SIgA-tagged microbiota using IgA-tagged magnetic beads, and to characterize the composition of SIgA-tagged microbiota of oral secretion and rumen.

## Materials and Methods

### Animals and Sampling

The animal study was approved by the Animal Care and Use committee of the University of Alberta according to the guidelines of the Canadian Council on Animal Care (Canadian Council on Animal Care [CCAC], 2009) at the Dairy Research and Technology Centre (Edmonton, AB, Canada). A total of six ruminally cannulated lactating Holstein dairy cows (4.5 ± 1.4 years) were used in this study. Four of the six animals were sampled twice while fed different dietary treatments at two periods to be considered individual experimental units (*n* = 10). Rumen contents collected from grab samples of the entire rumen via cannula were pooled for each animal. Oral secretion samples (50 ml) were collected by inserting surgical tubing into the distal cheek and allowing saliva, mixed with bolus particulate to flow into sterile tubes, representing a pooled sample of parotid, submaxillary, sublingual, buccal and gingival salivary secretions and bolus particulate. Rumen contents and oral secretions were flash frozen in liquid nitrogen and stored at -80°C prior to analysis.

### Secretory IgA Quantification

Oral secretions were analyzed for SIgA concentration using a bovine specific IgA ELISA kit (Bethyl Laboratories, Inc., Montgomery, TX, USA) according to manufacturer’s instructions.

### Separating IgA Labeled Bacteria

#### Anti-secretory IgA Labeled Beads

To isolate bacteria bound to SIgA, magnetic beads were conjugated with anti-secretory IgA antibodies. Anti-secretory IgA beads were prepared using Pure Proteome^TM^ NHS flexi bind magnetic beads (EMD Millipore, Inc.). Briefly, 100 μL of suspended beads was transferred to a sterile 1.5 mL microfuge tube into a magnetic stand to remove storage buffer. The isolated beads were removed from magnetic stand and re-suspended in 500 μL of chilled 1 mM HCl equilibrium buffer. The re-suspended beads were placed back into the magnetic stand and the equilibration buffer was removed. Anti-secretory IgA antibodies (60 μL, 2 ng/ml) (Bethyl Laboratories, Inc.) were added to the beads and incubated for 2 h at room temperature with constant shaking. A subset of magnetic beads was kept separate and not conjugated with IgA to be used as a control for unspecific binding of bacteria to magnetic beads. The entire protocol, including the addition of samples and elution step, was continued as described below with this control. After incubation the antibody solution was removed by placing the tube back into the magnetic stand and pipetting off supernatant. Beads were washed four times with 500 μL of quench buffer (100 mM Tris-HCl, 150 mM NaCl, pH 8.0). On the last wash the beads were incubated with quench buffer for 1 h at room temperature to block free binding domains on the beads. Quench buffer was then removed and beads were re-suspended in 100 μL sterile 1× PBS, pH 7.4 and stored at 4°C.

#### Isolating SIgA Tagged Bacteria

To isolate SIgA-tagged bacteria from non-tagged bacteria the prepared anti-secretory IgA conjugated beads were used. The PBS buffer was removed from labeled-beads (50 μL) using a magnetic stand and pipetting off the supernatant. Beads were washed once in 500 μL sterile 1× PBS. The wash solution was removed using the magnetic stand and beads were re-suspended in 500 μL of oral secretions or rumen contents. Sample-bead mixture was incubated at room temperature for 2 h with constant shaking. After incubation beads were placed back in the magnetic stand to remove unconjugated sample. Beads were washed 5× in 500 μL of 1× PBS. After washing, beads were re-suspended in 150 μL of elution buffer (0.2 M Glycine, HCl pH 2.0). To collect SIgA-tagged bacteria, beads were placed back into the magnetic stand and the elution buffer was collected into a microfuge tube and quenched with an equal volume of 1 M NaOH. The elution step was repeated once to collect a total of 300 μL of eluent. To ensure the elution step was adequate to remove all SIgA-tagged bacteria from IgA conjugated beads, the remaining beads were re-suspended in 1x PBS for DNA isolation.

### DNA Isolation

For microbial composition, DNA was isolated from oral secretion, whole rumen, and SIgA-tagged oral secretion and SIgA-tagged rumen samples with the Pure Link^®^Genomic DNA kit as per manufacturers instructions (ThermoFischer Scientific, Grand Island, NY, USA). DNA was quantified using a Nano Drop spectrophotometer (Thermo Fisher Scientific, Inc., Ottawa, ON, Canada). DNA extraction from the eluent of non-conjugated magnetic beads resulted in undetectable amounts of DNA, providing evidence that no unspecific binding of bacteria to the magnetic beads occurred. The re-suspended magnetic beads post elution also resulted in undetectable quantities of DNA, verifying the wash protocol was sufficient to remove all IgA-tagged bacteria from the anti-secretory IgA conjugated magnetic beads.

### Sequencing of 16S rDNA Gene

Regions V1–V3 of the bacterial 16S rDNA gene were amplified with nucleotide-barcoded primers 27F: 5′-AGAGTTTGATCMTGGCTCAG-3′ and 519R: 5′-GWATTACCGCGGCKGCTG-3′ (Lane, 1991). The 27F primer contained Roche/454 Titanium adaptor A (CCATCTCATCCCTGCGTGTCTCCGACTCAG) and unique 10-bp barcodes, the 519Rprimer contained adaptor B (CCTATCCCCTGTGTGCCTTGGCAGTCTCAG) (Lane et al., 1985). DNA was amplified in 20 μl reaction volumes containing 0.2 μl Phusion high-fidelity DNA polymerase (ThermoFischer Scientific.), 4 μl of 5× HF buffer, 0.4 μl 10 mM dNTPs, 1 μl of the extracted template DNA (100 ng), and 1 μl each F27 and R519 primers (10 ng/μl). The PCR parameters were: 98°C for 1 min, 35 cycles of 98°C for 10 s, 59°C for 30 s and 72°C for 30 s, with a final extension at 72°C for 7 min (S1000 Thermo Cycler, Bio-Rad, Hercules, CA, USA). Following amplification, products were pooled and run at 100 V for 1 h on a 0.8% agarose gel (SYBR Safe stain, Invitrogen). Bands corresponding to bacterial 16S rDNA gene were excised and gel-purified (QIAquick gel extraction kit, Qiagen, Valencia, CA, USA). Amplicons with sufficient DNA (100 ng) were pooled and pyrosequenced using a 454 Titanium platform (Roche, Branford, CT, USA) (*n* = 10/saliva, *n* = 9/rumen, *n* = 6/Saliva SIgA, *n* = 5/Rumen SIgA).

### Sequence Analysis

DNA sequencing results were analyzed using the Mothur pipeline (Schloss et al., 2009). Briefly, sequences were trimmed of barcodes and primers and sequences containing ambiguous bases, chimeras and quality read lengths < 200 bases were discarded (maximum ambiguous bases = 0, maximum homopolymers = 8, qwindowaverage = 30, and qwindowsize = 50). Subsequently, remaining sequences were aligned to SILVA (Quast et al., 2013). Sequences were clustered into operational taxonomic units (OTUs) based on 97% similarity using uClust algorithm. Sequencing yielded 1164 ± 162 sequences per sample that passed quality filtration. Samples failing to have a read depth of 728 were removed from downstream analysis, and all samples were normalized to 728 reads.

### Statistical Methods

Results are presented as means ± SEM. Normality of all variables was tested using Proc univariate of SAS (SAS version 9.3). In the instance of non-normally distributed data, values were subject to square root or log_10_+1 transformations. Data were analyzed by the univariate analysis of variance to examine associations between variables. Comparisons of treatments were determined by contrast of target groups (SAS^®^Studio, University Edition). To depict microbial community composition between oral secretion and whole rumen samples, and IgA labeled vs. whole samples, non-parametric multidimensional scaling (NMDS) ordination plots were generated using Euclidean distances in PAST (v3.11, Ø. Hammer 1999–2016). An analysis of similarities (ANOSIM) was used to determine differences between community compositions. Significance was set as *P <* 0.05. The raw sequences were deposited in the National Center for Biotechnology Information (NCBI) Sequence Read Archive (SRA) under the accession no. SRP101656.

## Results and Discussion

### Secretory IgA in Saliva

It has been demonstrated that SIgA is a significant component of saliva, representing greater than 85% of total immunoglobulin’s (Lascelles and McDowell, 1970; Mach and Pahud, 1971). It was previously found that salivary IgA concentration was approximately 0.56 mg/ml for Simmental cattle (Mach and Pahud, 1971), which is significantly lower than the concentrations measured in the current study (5.95 ± 1.21 mg/ml). Certain secretory regions of the bovine mouth contain differing concentrations of SIgA (Lascelles and McDowell, 1974), potentially explaining why our mixed saliva sample had increased SIgA concentration than what was determined previously from buccal samples.

### Detecting SIgA-Tagged Microbiota

Recently, it has been established that bacteria can be grouped into SIgA-tagged vs. non IgA-tagged fractions in both mice (Palm et al., 2014; Bunker et al., 2015) and humans (Palm et al., 2014; Kau et al., 2015). Using fluorescence-activated bacterial cell sorting (FACS), followed by 16S rDNA amplicon sequencing, researchers have successfully characterized differences between SIgA-tagged vs. SIgA-non-tagged bacteria (Palm et al., 2014; Bunker et al., 2015; Kau et al., 2015). The multi-step FACS is a significant advancement from original use of targeted fluorescent *in situ* hybridization analysis combined with flow cytometer detection (Tsuruta et al., 2010, 2012). The novel methodology developed in the current study streamlines the fractioning of the SIgA-tagged microbiota from original whole samples, allowing for rapid sequencing eliminating the need for a multi-step procedure requiring a flow cytometer. One limitation of the study is that not all samples were successfully amplified, leaving *n* = 5 for rumen sIgA-tagged bacteria and *n* = 6 for saliva sIgA-tagged bacteria, however, it is expected that this challenge could be overcome with further optimization.

### Oral Secretion vs. Whole Rumen Microbial Composition

The development and implementation of massive parallel sequencing has increased viability of studying the complex host–microbial interactions in ruminants, getting us one step closer to being able to use the rumen microbiome in phenotypic selection criteria. Currently, rumen sampling is accomplished through oral incubation (Shen et al., 2012), rumenocentisis, or preferentially, cannulation (Lodge-Ivey et al., 2009). Recently it has been proposed that sampling of buccal fluid, saliva mixed with regurgitated digesta, may be used in lieu of invasive rumen sampling (Tapio et al., 2016). However, our sequence data revealed that there was a measurable difference between our oral secretion samples and whole rumen content microbial composition. It must be pointed out that differences in whole rumen and oral secretions of the current manuscript and those found by Tapio et al. (2016) may in part be due to differences in sampling technique. The current study oral secretions were obtained by inserting sterile tubing into the buccal region of the mouth, whereas Tapio et al. (2016) used a buccal swab.

**FIGURE 1:**
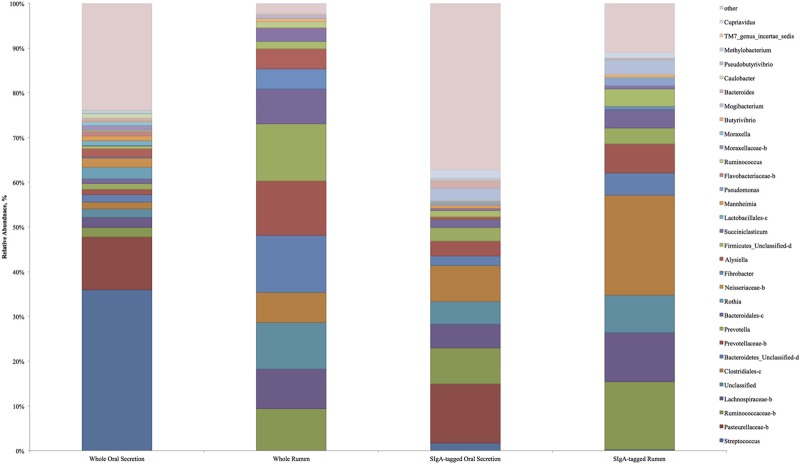
**Relative abundance of predominant microbial taxa in whole and SIgA-tagged oral secretion and rumen samples.** Letter behind taxonomy indicates unclassified: b – genus, c – family and genus, d – order, family and genus.

**FIGURE 2:**
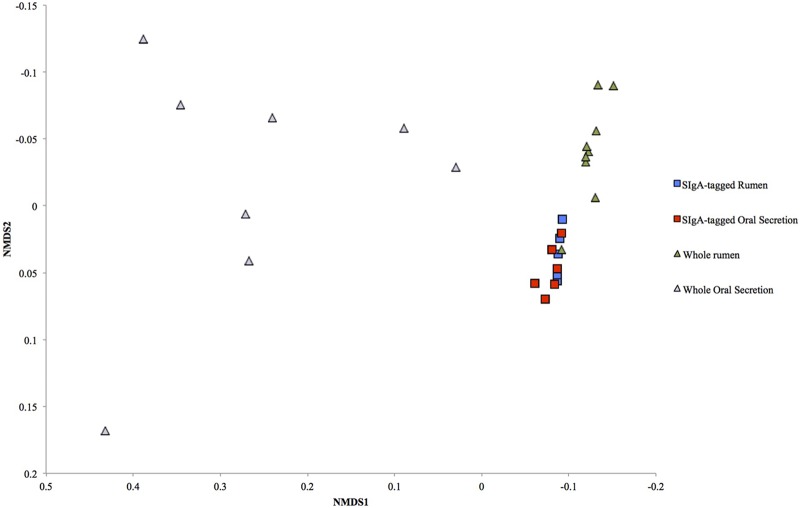
**Non-parametric multidimensional scaling (NMDS) ordination plot of bacterial communities.** The NMDS plot was generated using Euclidean distance metric between whole rumen, oral secretion, SIgA-tagged rumen, and SIgA-tagged oral secretion microbiota. Each dot represents an individual sample collected from whole rumen (*n* = 9), oral secretion (*n* = 10), SIgA-tagged rumen (*n* = 5), and SIgA tagged oral secretion (*n* = 6). The *R*^2^ for NMDS1 and NMDS2 axes were 0.94 and 023, respectively.

Due to low depth (<720 reads per sample) and below threshold sequence quality (30), one experimental unit was removed from whole rumen samples leaving *n* = 9 for whole rumen and *n* = 10 for saliva. In the current study Firmicutes, Bacteroidetes, members of Clostridia cluster XIVa (Ruminococcaceae and Lachnospiraceae), as well as *Prevotella* dominated rumen microbiota (**Table 1** and **Figure 1**), similar to previous reports (Hess et al., 2011; Tapio et al., 2016). Oral secretion microbial composition differed from that of whole rumen microbiota due to increased abundances of Firmicutes, Proteobacteria, Actinobacteria, and Fusobacteria (**Table 1**, *P <* 0.05). In contrast, whole rumen content had increased abundance of Bacteroidetes, Fibrobacter, and Tenericutes vs. oral secretion (*P* < 0.05). *Pseudomonas*, *Mogibacterium*, and *Cupriavidus* were the only similarly abundant taxa (*P* > 0.05) when comparing oral secretion and whole rumen samples. However, most predominant genera including *Prevotella*, and an unclassified genus of Prevotellaceae, were distinctively abundant between oral secretion and whole rumen samples (*P* < 0.05). The difference in community composition between the two sites was further indicated by the NMDS plot in **Figure 2** and corresponding Euclidean distances (**Table 2**). Thus our data strongly indicates that saliva and the rumen are substantially different micro-environments and that oral sampling is likely not a suitable proxy for rumenal digesta. However, this is in direct contradiction to previous work, which concluded buccal swabs would be an appropriate proxy to the rumen (Tapio et al., 2016).

**Table 1:** Relative abundance of predominant bacterial taxa and their *post hoc* comparisons.

Taxonomy	Oral secretion	Whole rumen	SIgA –tagged oral secretion	SIgA tagged SEM^a^ rumen		Oral secretion vs. whole rumen	SIgA-tagged oral secretion vs. IgA-tagged rumen	SIgA-tagged oral secretion vs. whole rumen	SIgA-tagged saliva vs. oral secretion
						
						*P-*values			
**Phyla**									
Firmicutes	50.75	34.99	37.44	63.12	0.187	0.016	0.007	0.580	0.102
Bacteroidetes	8.79	47.28	14.23	19.67	0.261	<0.001	0.268	<0.001	0.218
Proteobacteria	28.61	0.39	29.55	3.93	0.514	<0.001	0.001	<0.001	0.987
Actinobacteria	3.54	0.01	1.14	0.23	0.092	<0.001	0.242	0.029	0.087
Fibrobacteres	0.17	4.33	0.08	0.41	0.167	<0.001	0.163	<0.001	0.480
Fusobacteria	1.04	0.02	2.27	0.28	0.036	0.005	0.020	<0.001	0.250
TM7	0.11	0.22	0.12	0.11	0.104	0.401	0.945	0.510	0.938
Tenericutes	0.02	0.33	0.02	0.01	0.024	0.019	0.970	0.029	0.762
**Genera**									
*Streptococcus*	35.98	0.01	1.73	0.30	0.311	<0.001	0.313	0.073	<0.001
Pasteurellaceae^b^	11.76	0.00	13.20	0.00	0.544	<0.001	<0.001	<0.001	0.671
Ruminococcaceae^b^	2.13	9.43	8.06	15.16	0.121	<0.001	0.032	0.565	0.001
Lachnospiraceae^b^	2.31	8.80	5.35	10.96	0.101	<0.001	0.027	0.078	0.037
Clostridiales^c^	1.62	6.66	8.08	22.33	0.136	<0.001	<0.001	0.549	<0.001
Bacteroidetes_Unclassified^d^	1.55	12.78	2.09	4.95	0.101	<0.001	0.052	<0.001	0.636
Prevotellaceae^b^	1.28	12.24	3.32	6.56	0.092	<0.001	0.108	<0.001	0.074
*Prevotella*	1.29	12.69	3.03	3.46	0.131	<0.001	0.953	<0.001	0.225
Bacteroidales^c^	0.98	7.87	1.72	4.10	0.070	<0.001	0.049	<0.001	0.313
*Rothia*	2.62	0.00	0.06	0.10	0.171	<0.001	0.880	0.565	0.005
Neisseriaceae^b^	2.06	0.00	0.09	0.00	0.096	<0.001	0.487	0.425	0.003
*Fibrobacter*	0.18	4.46	0.11	0.66	0.035	<0.001	0.067	<0.001	0.470
*Alysiella*	1.90	4.46	0.45	0.01	0.035	<0.001	0.120	0.075	0.010
Firmicutes_Unclassified^d^	0.60	1.63	1.36	3.96	0.059	0.046	0.013	0.733	0.146
*Succiniclasticum*	0.08	3.03	0.47	0.68	0.052	<0.001	0.645	<0.001	0.146
Lactobacillales^c^	1.13	0.00	0.08	0.00	0.065	<0.001	0.504	0.325	0.009
*Mannheimia*	1.09	0.02	0.69	0.00	0.064	<0.001	0.027	0.006	0.429
*Pseudomonas*	0.01	0.00	0.58	1.76	0.185	0.840	0.336	0.142	0.185
Flavobacteriaceae^b^	0.99	0.00	0.00	0.01	0.033	<0.001	0.666	0.620	<0.001
*Ruminococcus*	0.26	1.39	0.19	0.26	0.036	0.002	0.758	0.002	0.723
Moraxellaceae^b^	1.06	0.00	0.04	0.00	0.017	<0.001	0.304	0.239	<0.001
*Moraxella*	0.82	0.00	0.04	0.00	0.029	<0.001	0.397	0.331	<0.001
*Butyrivibrio*	0.24	0.69	0.12	0.54	0.032	0.032	0.122	0.007	0.357
*Mogibacterium*	0.01	0.00	2.75	3.03	0.055	0.802	0.793	<0.001	<0.001
*Bacteroides*	0.60	0.04	1.79	0.06	0.081	0.030	0.006	0.002	0.149
*Caulobacter*	0.92	0.05	0.06	0.07	0.047	<0.001	1	0.798	<0.001
*Pseudobutyrivibrio*	0.16	0.79	0.01	0.28	0.021	0.002	0.032	<0.001	0.075
*Methylobacterium*	0.56	0.04	0.34	0.11	0.076	0.005	0.406	0.341	0.106
*Cupriavidus*	0.06	0.00	1.81	1.09	0.060	0.318	0.303	<0.001	<0.001


*^a^SEM, pooled standard error of the mean;*

*^b^Unclassified genus;*

*^c^Unclassified family and genus;*

*^d^Unclassified order, family and genus.*

**Table 2:** Pairwise-comparison of Euclidean distances between whole and IgA-tagged rumen and saliva samples.

	SIgA-tagged rumen		SIgA-tagged oral secretion		Whole rumen	
			
	*P-*value	*R-*value^a^	*P-*value	*R*-value	*P-*value	*R*-value
SIgA-tagged rumen	–	–	–	–	–	–
SIgA-tagged oral secretion	0.349	0.024	–	–	–	–
Whole rumen	0.001	0.689	<0.001	0.710	–	–
Oral secretion	0.015	0.412	0.006	0.449	<0.001	0.635


*^a^*R*-value, Euclidean distance between samples.*

### Secretory-IgA Microbial Targeting

**FIGURE 3:**
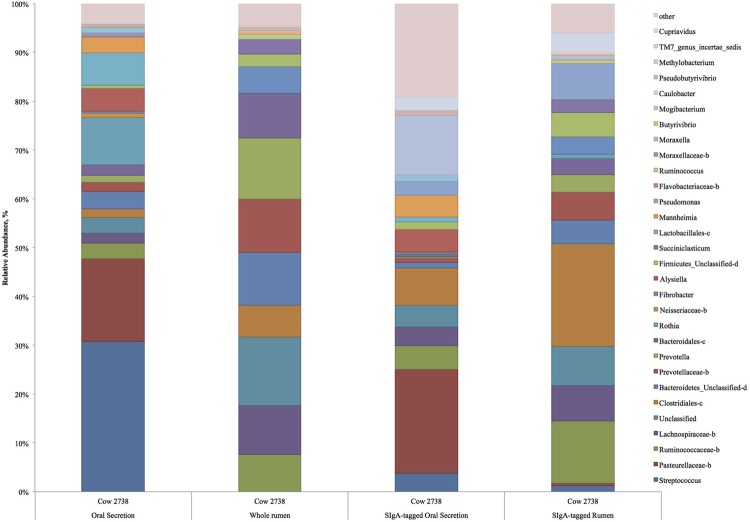
**Relative abundance of predominant taxa in whole and IgA-tagged oral secretions and rumen samples of individual Cow 2738.** Letter behind taxonomy indicates unclassified: b – genus, c – family and genus, d – order, family and genus.

Although there were distinct differences in microbial community composition between whole rumen and oral secretion samples, our data revealed a measurable similarity between SIgA-tagged oral secretion and SIgA-tagged rumen microbial composition, suggesting specific targeting may be occurring. Specific SIgA targeting has previously been demonstrated in mice with SIgA-tagged taxa abundant in both duodenum and colon (Bunker et al., 2015). After discarding samples with below threshold quality scores and low reads per sample resulted in *n* = 5 for rumen sIgA-tagged bacteria and *n* = 6 for oral secretion sIgA-tagged bacteria. The phyla, Bacteroidetes, Actinobacteria, Fibrobacteres, candidate phyla TM7, and Tenericutes were analogous (**Table 1**, *P* > 0.05) between SIgA-tagged saliva and SIgA-tagged rumen samples. Although there was a high degree of similarity between the SIgA-tagged saliva and SIgA-tagged rumen samples the relative abundance of certain taxa were significantly different between the two including unclassified genera of Pasteurellaceae, Ruminoccaceae, Lachnospiraceae, an unclassified family and genus of Clostridiales and Bacteroidales, an unclassified class of Firmicutes, and genera *Mannheimia, Bacteroides*, and *Pseudobutyrivibrio* (**Table 1**, *P* < 0.05). A limitation of the study is that we were not able to compare bacterial composition of the each animal individually because not all samples provided sufficient DNA or sequencing depth. One animal where all four samples were successful is depicted in **Figure 3**, and shows the same patterns as those seen for the entire data set, where SIgA preferentially binds selected bacteria in both the rumen and saliva.

To strengthen the claim SIgA targets commensal microbiota, we evaluated the phylogenetic similarities in microbial composition between saliva and rumen whole and SIgA-tagged using a NMDS plot via the Euclidean distance metric. Based on the NMDS plot, community composition was only similar between SIgA-tagged bacteria from saliva and the rumen (**Figure 2** and **Table 2**, ANOSIM; *P* > 0.05). The high degree of similarity in community composition between SIgA-tagged saliva and SIgA-tagged rumen microbiota may indicate a commensal bacteria selective mechanism, as these SIgA-tagged microbiota are retained within the rumen. When mice lacking a functional adaptive immune system are colonized with single species of bacteria they display an increased innate immune response in comparison to mice with functional IgA (Peterson et al., 2007). This suggests the role of IgA is to mediate immune tolerance and homeostasis, allowing the host to detect new bacteria while ignoring the presence of those previously encountered (Peterson et al., 2007). Evidence from recent studies indicates that SIgA-coating is a selective mechanism for commensal bacteria, supporting both pathogenic and commensal colonization, dependent on originating host health status (Palm et al., 2014; Kau et al., 2015). In a pre-existing dysbiotic state SIgA can selectively coat known inflammatory and pathogenic bacteria (Palm et al., 2014), disrupting intestinal barrier function causing weight loss and sepsis when transplanted into gnotobiotic animals (Kau et al., 2015). However, administration of SIgA-targeted bacteria from a healthy microbiome was found to prevent a disease phenotype from occurring (Kau et al., 2015). The fact that SIgA binds many native rumen bacteria and these ‘tagged’ bacteria remain detectable within the rumen may indicate salivary SIgA is acting as a selection tool for commensal colonization in a healthy state.

### Whole Rumen vs. Saliva SIgA-Tagged Microbial Composition

Interestingly, the SIgA-tagged oral secretion microbiota more closely resembled whole rumen microbial composition vs. whole oral secretion due to enrichment and depletion of specific bacteria. This further implies SIgA-coating of microbiota may be a commensal selection mechanism. The unclassified genera of Lachnospiraceae, unclassified family of Clostridiales, and an unclassified class of Firmicutes were all enriched proportionally in the salivary SIgA-tagged microbiota vs. oral secretion, resulting in them becoming statistically similar to whole rumen (*P* > 0.05). Other bacteria including phylum Firmicutes, genus *Streptococcus*, *Rothia*, unclassified genus of Neisseriaceae, unclassified family of Lactobacillales, unclassified genus of Moraxellaceae, genus *Moraxella*, *Caulobacter*, and *Methylobacterium* were depleted in salivary SIgA-tagged microbiota vs. oral secretion, causing the microbial composition to have an increased resemblance to whole rumen microbiota (*P >* 0.05). An unclassified genus of Flavobacteriaceae also became non-detectable in salivary IgA-tagged microbiota vs. oral secretion, further increasing its similarity to whole rumen samples (*P* > 0.05). Vast differences in microbial composition between SIgA-tagged bacteria in the saliva with that of oral secretion further indicate the SIgA is targeting rumen microbiota over saliva microbiota. Of the microbiota listed *Streptococcus*, Ruminococcaceae, Lachnospiraceae, Clostridiales, *Rothia*, Neisseriaceae, *Alysiella*, Lactobacillales, Flavobacteriaceae, Moraxellaceae, *Moraxella*, and *Caulobacter* were all non-differing between SIgA-saliva and whole rumen; however, significantly different between Saliva and SIgA-saliva.

## Conclusion

Understanding the mechanisms behind host-derived selection of commensal microbiota in ruminants is increasingly necessary to enable us to use microbial phenotypes as a selection tool improving feed conversion and health. In the current study our immune-affinity based benchtop method allowed for rapid separation of SIgA-tagged bacteria and microbial characterization via next generation sequencing. Large compositional differences between oral secretion and rumen microbial profiles do not support the use of saliva as a proxy for the rumen contents. SIgA-tagging resulted in the enrichment of Ruminococcaceae, Lachnospiraceae, Clostridiales, and unclassified Firmicutes within the saliva, resulting in SIgA-tagged saliva microbiota to more closely resemble whole rumen microbiota vs. oral secretion. Thus, saliva SIgA-coating of microbiota within a healthy host may be a potential host-derived adaptive immune mechanism for rumen commensal colonization. Future work to explore the cross-reactivity of salivary SIgA between animals will help define the role of SIgA in host specificity.

## Author Contributions

Conceived and designed the experiments: LG, BW. Performed the experiments: LS, MT. Analyzed the data: JF, BW. Wrote the paper: JF, BW.

## Conflict of Interest Statement

The authors declare that the research was conducted in the absence of any commercial or financial relationships that could be construed as a potential conflict of interest.
